# The effect of hard tissue defects on the clinical outcome of endodontic microsurgery: a systematic review and meta-analysis

**DOI:** 10.1007/s00784-023-05341-3

**Published:** 2023-11-07

**Authors:** Mohammad Sabeti, Mohammad Saqib Ihsan, Piyusha Kharat, Amir Azarpazhooh

**Affiliations:** 1 Advanced Specialty Program in Endodontics, UCSF School of Dentistry, 707 Parnassus Ave. Room- D 3226, San Francisco, CA 94143-0758 USA; 2 UCSF Advanced Specialty Program in Endodontics, 707 Parnassus Ave. Room- D 3226, San Francisco, CA 94143-0758 USA; 3 UCSF, School of Dentistry, 707 Parnassus Ave, San Francisco, CA 94143-0758 USA; 4 University of Toronto, Faculty of Dentistry, 455-124 Edward St, Toronto, ON M5G1G6 Canada

**Keywords:** Surgical endodontics, Systematic review, Prospective cohort, Meta-analysis, Evidence-based dentistry

## Abstract

**Objectives:**

The purpose of this systematic review was to appraise the existing literature on the effect of hard tissue defects on the clinical outcome of endodontic microsurgery (EMS).

**Methods:**

MEDLINE (PubMed), Embase, Web of Science, Cochrane Library and grey literature were searched from January 2000 to May 2023. Study selection and data extraction were performed in duplicate. Eligible studies were critically appraised for the risk of bias using the Cochrane Risk of bias tool. The quality of evidence was assessed using GRADE. Review Manager (RevMan Computer program Version 5.4, The Cochrane Collaboration, 2020) was utilized and the Mantel Haenszel fixed or random effects model was applied, depending on the heterogeneity of the studies. Meta-analysis was performed to estimate the Risk ratio (RR) and 95% Confidence Interval (CIs) to correlate the effects of these factors on treatment outcomes.

**Results:**

Nineteen studies were included. The EMS overall pooled success rate was 84.5%. Five characteristics of hard tissue were identified. The size of the lesion (Small ≤ 5 mm: 78.4% vs. Large > 5 mm: 63.3%, RR = 1.12, 95% CI 1.00–1.26, *P* ≤ *.05*), significantly affected the outcomes of EMS. Endodontic lesions exhibited slightly better outcomes than endodontic-periodontal lesions (81.4% vs. 68.2%, RR = 1.14 95% CI 0.98–1.33, P > *.05*). Cases with the height of the buccal bone > 3 mm also exhibited slightly better outcomes (91.5% vs. 71.4%, RR = 1.20, 95% CI 0.88–1.62, P > *.05*). Additionally, through and through lesions exhibited better outcomes when grafting was completed during the EMS procedure both in 2D (RR = 1.12 95% CI 0.97–1.29, P > *.05)* and 3D evaluation ((RR = 1.28 95% CI 0.69–2.37 P > *.05*). The overall quality of evidence was graded as low to high.

**Conclusion:**

With a low to high quality of evidence, the size of the lesion is a key prognostic variable that significantly affects the outcome of EMS, as lesions ≤ 5 mm exhibit better outcomes as compared to larger lesions.

**Clinical significance:**

The presence of hard tissue defects can affect the outcome of endodontic microsurgery (EMS). The presented data can aid the clinicians’ decision-making process by examining certain pre-operative prognostic variables, when considering EMS as a treatment option. Clinical cases with more favorable hard tissue characteristics lead to a better prognosis in EMS.

## Introduction

Endodontic microsurgery (EMS) procedures are indicated in previously treated teeth where non-surgical retreatment is not feasible or as an adjunct to non-surgical root canal treatment in teeth with longstanding periapical disease and can also be useful in the diagnosis of pathosis [1]. The primary objective of EMS is to restore the apical periodontium to its functional state [2, 3]. EMS success depends on the absence of clinical symptoms, such as pain, swelling, tenderness to percussion, lack of sinus tracts, and normal, physiologic tooth mobility [4]. Various preoperative and postoperative factors can differ widely in magnitude at different points in time, making it difficult to assess their combined effects on the outcome of treatment [5].

In recent decades, numerous clinical studies have focused on the technical aspects of EMS, particularly on the innovative use of instruments, devices, and filling materials [6]. Subsequently, improvements in techniques, magnification aids, and the use of biocompatible root-end filling materials have made EMS treatment outcomes more predictable [7]. In a meta-analysis, the weighted pooled success rate of EMS was 95% (95% confidence intervals (CI), 0.88–0.98), making it 1.6 times more successful than traditional surgery, which had a weighted pooled success rate of 59% (95% CI, 0.55–0.63) [8].

Following EMS, soft tissue incisions heal mainly by primary intention, whereas bone defects and resected root surfaces heal by secondary intention [9]. The periapical pathosis, bone tomography, lesion size and inadequate bone preparation during osteotomy procedures can, however, affect the quality, quantity, and level of bone destruction, leading to delayed healing and a higher risk of postoperative complications [10]. The characteristics of hard tissues, and their effect on EMS outcomes have not yet been comprehensively analyzed. The presence of multiple unfavorable hard tissue characteristics may lead to a poor EMS prognosis. Sufficient knowledge about the characteristics of hard tissues can aid the clinician in the development of the treatment plan when considering it as a treatment option for the healing of persistent apical pathosis. Therefore, the purpose of this systematic review was to determine whether the presence of hard tissue defects influenced outcome of treatment in patients who had undergone EMS with a minimum one-year follow-up period.

## Materials and methods

This systematic review was registered in the PROSPERO database (CRD42021270431) under the universally accepted systematic review process [11]. The Preferred Reporting Items for Systematic Reviews and Meta-Analyses (PRISMA) statement was used to report our methodology and results [12].

The eligibility and criteria were as follows:

- *Population:* Adults with a history of the previously treated teeth with EMS. For the purposes of this review, EMS procedures should include the use of a surgical operating microscope, ultrasonic root-end preparation, and biocompatible root-end filling materials.
- *Exposure*: Hard tissue prognostic variables such as the size of lesion, type of lesion (presence of endodontic-periodontal), the use of grafting in through and through lesions (TATL), and the height of the buccal bone plate, during EMS.
- *Outcome (O):* The outcomes of EMS in each study were evaluated clinically and/or radiographically, according to Rud et al. [13] and Molven et al. [14] classifications, or modified PENN 3D criteria [15], namely, complete healing (reformation of the lamina dura), incomplete healing (scar tissue), uncertain healing, and unsatisfactory healing. Success was defined as the absence of clinical signs and symptoms (pain, swelling, tenderness to percussion, sinus, and lack of mobility) and evidence of radiographic healing as determined by CBCT or PA (absence of periapical radiolucency). The criteria for failure included any clinical signs and/or symptoms or radiographic evidence of uncertain or unsatisfactory healing. The success of EMS should be measured after a period of at least 12 months.
- *Study design:* Randomized controlled trials, prospective and retrospective cohort studies were included. A prospective study design was selected because preoperative factors, outcome assessments, and follow-up examinations can be easily documented and analyzed.

Studies were excluded if they involved the primary dentition, teeth with horizontal or vertical fractures, studies in which the effect of hard tissues on the outcome of EMS was not examined, studies involving other surgical endodontic procedures (e.g., replantation, hemisection, root amputation), studies with inconsistent designs, non-endodontic microsurgical procedures, outdated retrograde materials (i.e. amalgam), studies with < 12 months follow-up periods, studies conducted on animal, ex vivo or in vitro studies, as well as review articles, letters, and opinion articles were also excluded.

### Search methods for identification of studies

Three independent reviewers were involved in determining the search terms (P.K., M.S., and M.S.I) in consultation with the librarian. Four major electronic databases were used: MEDLINE (PubMed), Embase, Web of Science and the Cochrane Library up to March 2023, for English language studies. (Table 1 ). Unpublished studies (or grey literature, such as technical reports or dissertations) through Google Scholar™ (Google, Mountain View, CA, USA) and ProQuest (Ann Harbor, Michigan, USA) (the first 100 hits) were also included. A reference list of previous reviews of the same topic, including studies and major textbooks [4, 5] was also manually searched. All identified records were retrieved and imported into bibliographic software (Zotero 5.0.83 version). Duplicate records were removed.

**Table 1:**
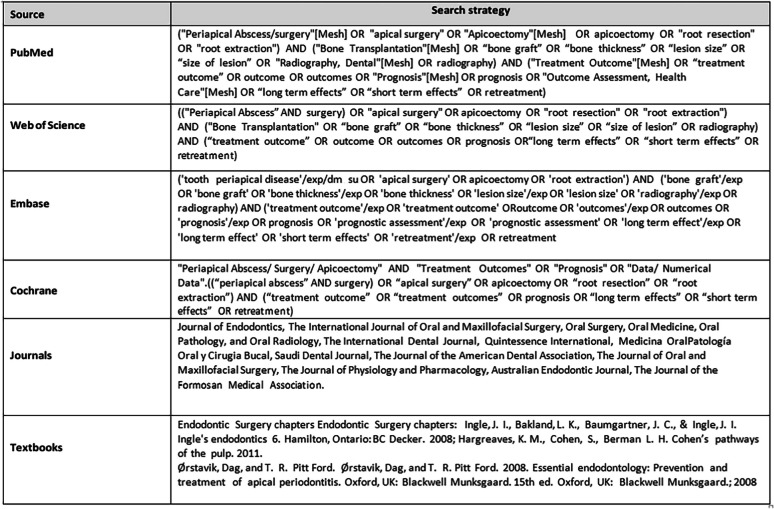
Search methodology

### Screening and data extraction

Two reviewers (P.K, M.S.I) independently reviewed and selected eligible studies from the searches and extracted the data. The disagreements were resolved by consulting a third reviewer (M.S), who reviewed the selection of studies and data extraction. The information outlined in Table 2 was extracted from each study to verify the inclusion criteria. The data from the included studies were entered into a standardized Excel (Microsoft, Richmond, WA, USA). Reasons for exclusion at the full-text stage were recorded (Table 3 ). The following data from each study was extracted:

- General characteristics of the study (author, year, title, type of study design)
- Detailed information about the participants, including preoperative pulpal and periapical diagnosis.
- A description of the exposure status and the number of subjects assigned to each prognostic factor of interest (lesion size, lesion type, height of buccal bone plate, and the duration of the follow-up visits)
- A description of the methods used to assess the outcomes.

Two independent reviewers (P.K and M.S.I) independently verified the accuracy of data extraction. The corresponding authors of all eligible studies were contacted when in doubt about the procedure, methodology, or material of the studies.

**Table 2:**
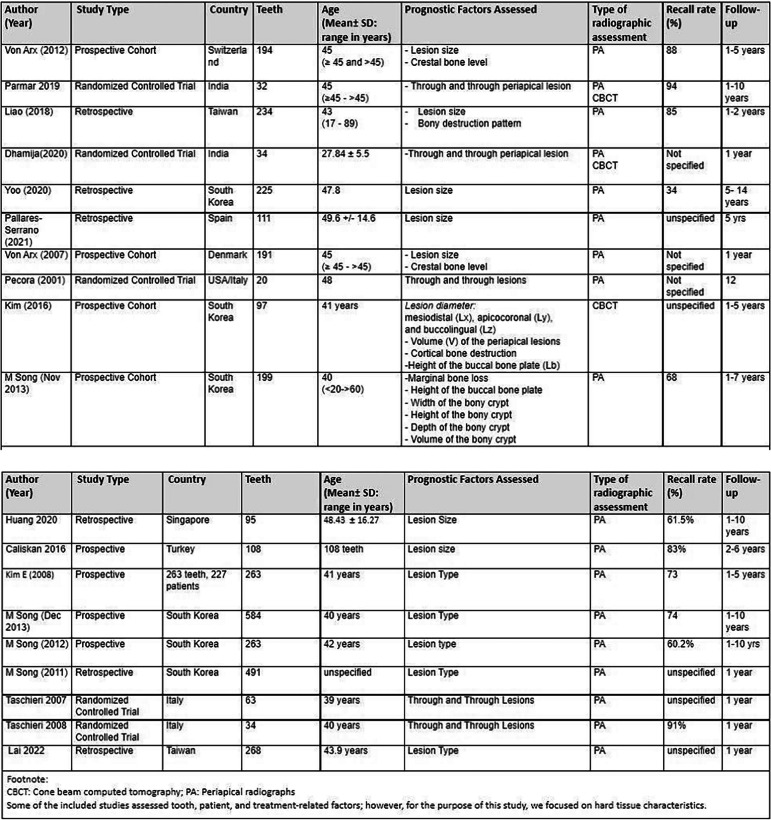
Characteristics of the included studies

**Table 3:**
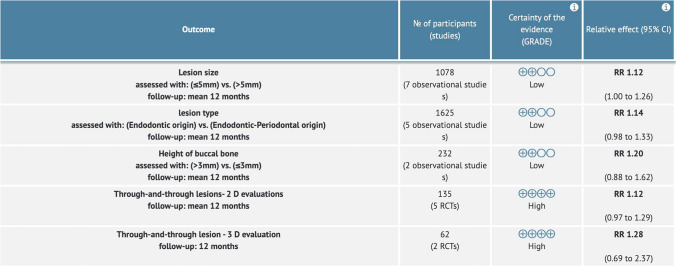
GRADE Assessment for the included studies

### Risk of bias assessment

Two authors (M.S.I and P.K) independently used the Cochrane risk of bias tool [16] to evaluate the included randomized controlled trials across 7 domains: random sequence generalization, allocation concealment, blinding of participants and personnel, blinding of outcome assessment, incomplete outcome data, selective reporting, and other bias. For retrospective and prospective studies, the items “random sequence generation”, “allocation concealment” and “blinding of participants and personnel” were rated as not applicable. Disagreements were resolved by consulting a third reviewer (M.S). A determination of low risk in all domains of a study rendered it as a “low risk” of bias, some concerns or unclear risk of bias in at least 1 domain classified the study as having “some concerns,” and some concerns in 3 or more domains or high risk in at least 1 domain rendered the study as “high risk.”

### Quality of the evidence

The Grading of Recommendations, Assessment, Development and Evaluation (GRADEpro GDT: GRADEpro Guideline Development Tool; McMaster University, Hamilton, ON, Canada) was used to objectively rate the quality of outcome analyses for the prospective and retrospective cohort studies. Two reviewers (P.K and M.S.I) assessed five categories: the risk of bias, inconsistent results, indirectness of the evidence, imprecision, and publication bias. (Table 3 ) An overall judgment of high, moderate, low, or very low confidence was given to each result [17]. Consensus was reached by consulting a third reviewer (M.S).

### Quantitative analyses

For each intervention, data was summarized, based on the outcomes associated with those teeth with or without the respective hard tissue characteristics. Based on the potential predictors, we stratified the subgroup analysis for the outcome of EMS during the follow-up period to further assess the following hard tissue characteristics: size and type of lesion, height of the buccal bone, and effect of grafting on outcome when through and through lesions were present. Review Manager (RevMan Computer program Version 5.4, The Cochrane Collaboration, 2020) [18] was utilized. Statistical heterogeneity was evaluated using the I^2^ statistic, with 25% equating low heterogeneity, 50% equating medium heterogeneity, and 75% equating high heterogeneity [16]. The Mantel Haenszel fixed-effects model of analysis was applied if the I^2^ was < 50% otherwise, a random-effects model of analysis was used [19, 20]. The meta-analysis used risk ratio (RR) and 95% CI to estimate outcome. Sensitivity analyses were performed to evaluate the influence of studies with a high risk of bias. For all test results, *P* ≤ *0.05* was considered significant.

## Results

### Search results

**Fig. 1:**
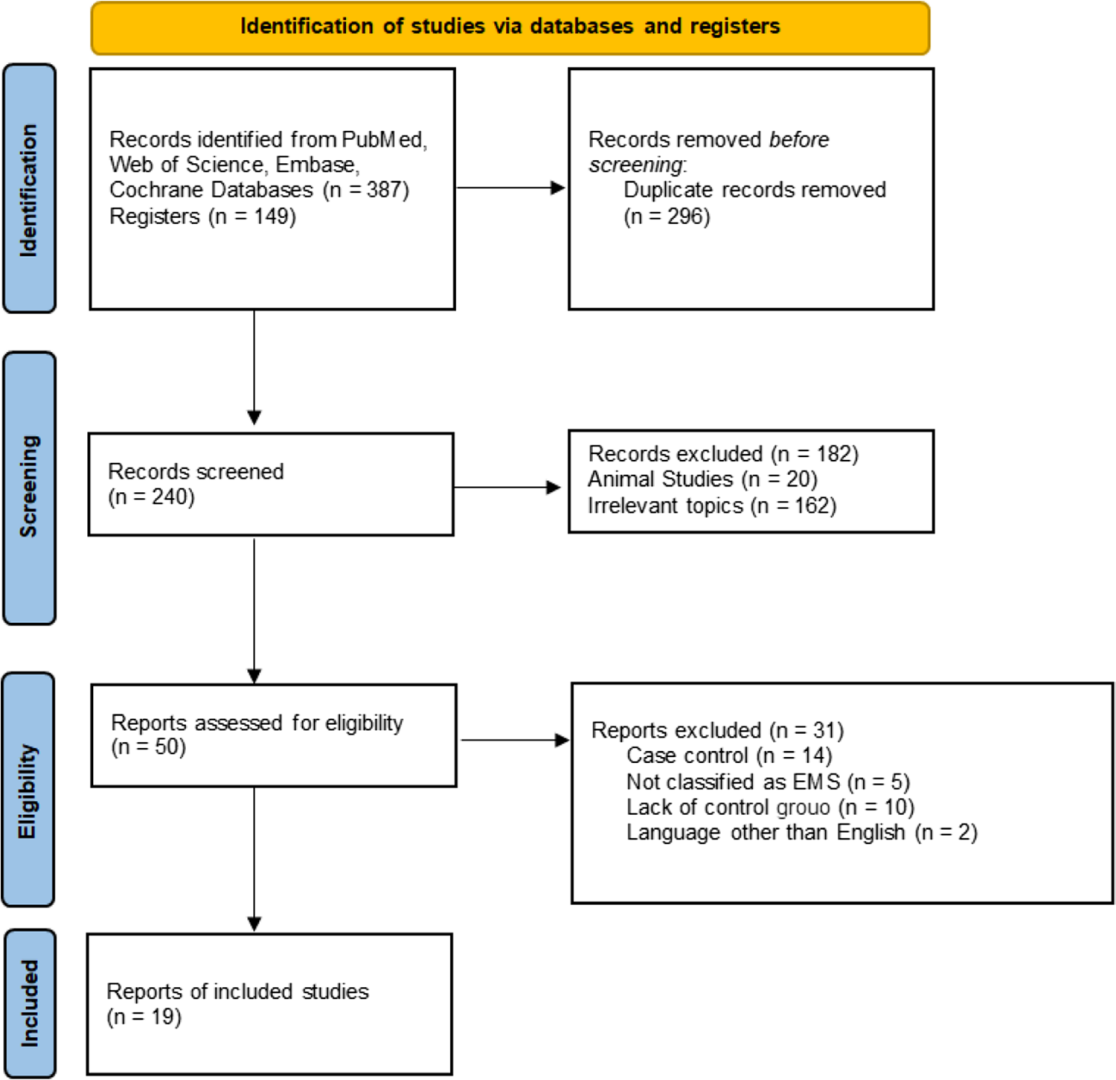
PRISMA Chart for study selection

From the initial search of 536 records, 296 duplicates were removed. The titles and abstracts of 240 records were screened. A total of 51 records were retrieved, and finally, 19 studies met our inclusion criteria [21–39] (Fig. 1). The reasons for exclusion can be found in the Supplementary Table.

### Descriptive results

The included studies (Table 2 ) were conducted in various locations. There were a total of 3,506 cases of EMS included. The decision to perform EMS was confirmed by the presence of either a radiographic apical radiolucency and/or clinical symptoms such as pain, tenderness to percussion, infection, and swelling of the buccal mucosa [21–39].

### Risk of bias

**Fig. 2:**
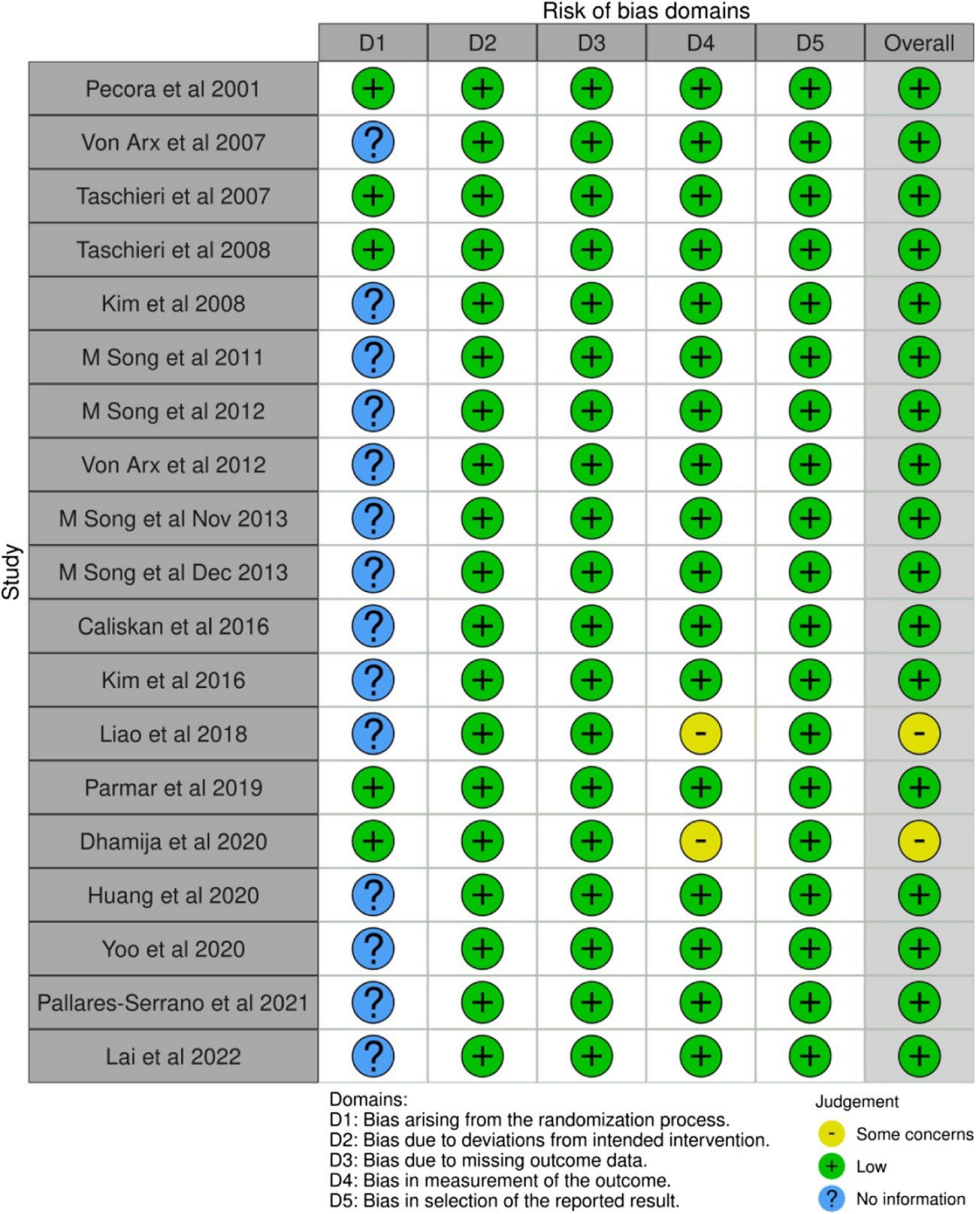
Risk of Bias for included studies

The results of the Cochrane Risk of Bias tool (RoB 2) is presented in Fig. 2. No included study was judged to exhibit a high risk of bias.

### Quality of evidence

The overall certainty of evidence across studies was rated as low to high. Due to the variation, this indicates that our confidence in the estimate of the effect is limited. Therefore, the true effect may be substantially different from the estimate of the effect. (Table 3).

### Quantitative analyses

**Fig. 3:**
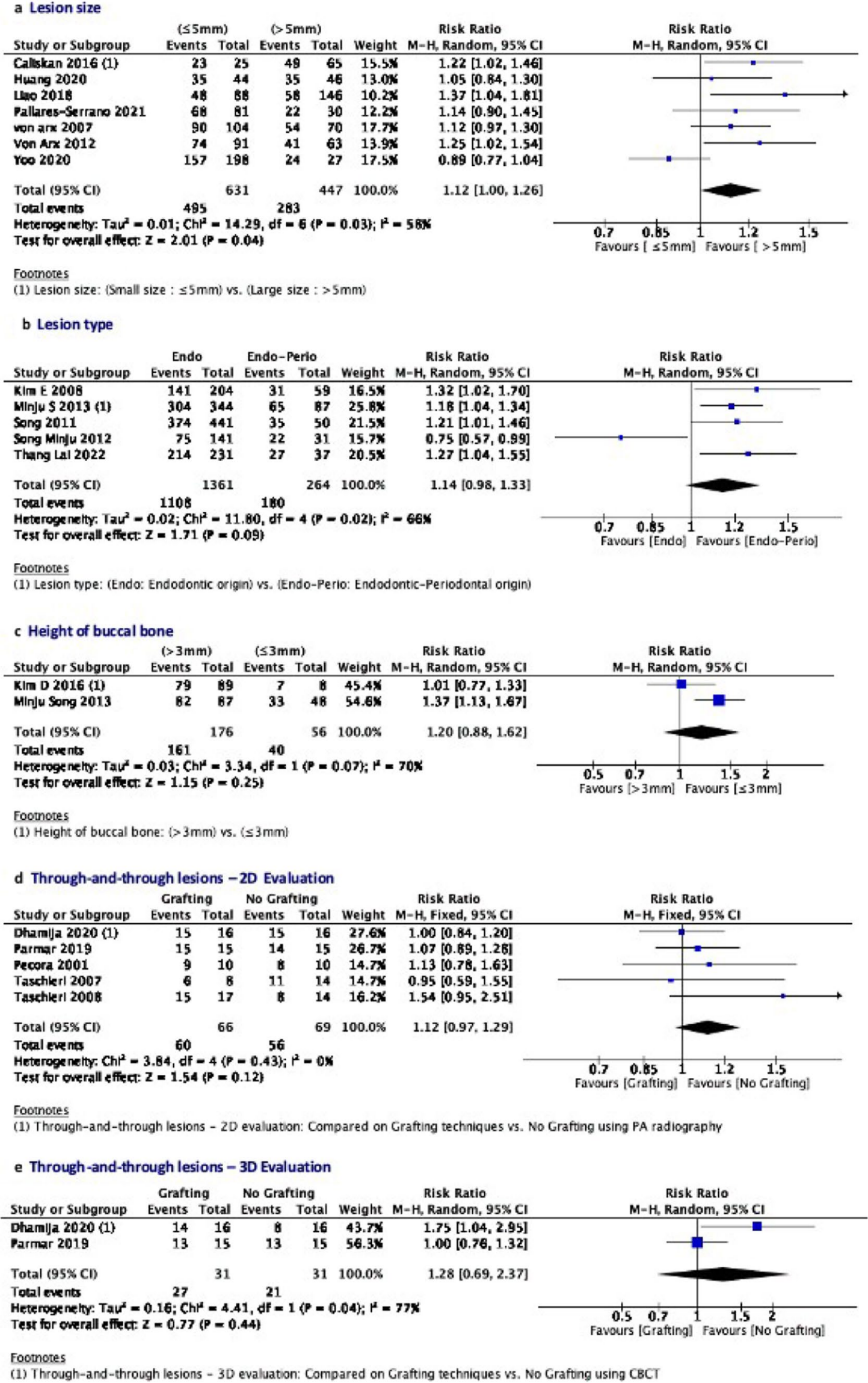
Forest plot of outcome comparison for all included studies

The overall pooled success rate of EMS from the 18 included studies is 84.5%**.** Meta-analysis was performed on each of the hard tissue characteristics (Fig. 3). The impact of the following hard tissue characteristics on the success of EMS outcome was investigated:

- *Lesion size:*
  o In seven studies [21–27], lesion size was measured by periapical radiographs and characterized as small (≤ 5 mm) or large lesions (> 5 mm). The pooled result of 1078 cases revealed that small lesions (≤ 5 mm) exhibited significantly better outcome of healing compared to large lesions (> 5 mm); (78.4% vs. 63.3%, RR = 1.12, 95% CI 1.00–1.26, *P* ≤ *0.05*). Medium heterogeneity was observed (I^2^ = 58%).
- *Lesion type:*
  o In five studies [28–32], the lesion type was dichotomized as endodontic vs. endodontic-periodontal lesion. The pooled result of 1,625 cases revealed that endodontic lesions exhibited slightly better outcomes of healing compared to the Endodontic-Periodontal lesion: (81.4% vs. 68.2%, RR = 1.14 95% CI 0.98–1.33, P > *0.05*). A medium level of heterogeneity (I^2^ = 66%) was observed.
- *Through and through lesions:*
  o In five studies [33–37], the outcome of through and through lesions was dichotomized, based on if grafting was used during the EMS procedure. The pooled result of 135 cases examined the result of EMS in the presence of through and through lesions and compared groups based on whether or not a graft was placed during the procedure. All the studies assessed the result using conventional PA radiography, but only two studies [35, 36] used CBCT as an adjunct. Both the 2D evaluation (RR = 1.12 95% CI 0.97–1.29, P > *0.05)* and the 3D evaluation (RR = 1.28 95% CI 0.69–2.37 P > *0.05*) showed that there were slightly better outcomes when a graft was utilized. There was no heterogeneity between the five studies, but a high level of heterogeneity was observed in the two 3D analysis studies (I^2^ = 77%).
- *Height of Buccal Bone:*
  o In two studies [38, 39], the height of buccal bone was dichotomized as ≤ 3 mm vs. > 3 mm. The pooled result of 232 cases revealed that those with a longer buccal bone height (> 3 mm) had slightly better outcomes compared to those with a shorter buccal bone height (≤ 3 mm); (91.4% vs. 71.4%, RR = 0.84, 95% CI 0.62–1.14, P > *0.05*). A medium-level heterogeneity was observed (I^2^ = 70%).

## Discussion

In this study, we assessed the effects of hard tissue defects on the outcome of EMS. With a low to moderate certainty, this systematic review and meta-analysis showed that the size of the lesion can significantly impact the healing outcomes of EMS. No major treatment-related concerns were reported in the included studies.

The success of EMS can be influenced by patient-related, tooth-related, and treatment-related factors [8]. In terms of tooth-related factors, the outcome of EMS can vary depending on the size of the lesion. Similar to previous research studies [8, 24, 40, 41], our study found that the size of the periapical lesion can affect the outcome of EMS. Our results were similar to other studies that assessed lesion size and outcome [24, 40, 41]. A systematic review that evaluated tooth-related prognostic factors when performing EMS found that, at a minimum follow-up period of 12 months, the healing rate was significantly higher for teeth with smaller lesions (≤ 5 mm) than for those with larger lesions (> 5 mm) (OR = 1.82; 95% CI, 1.13–2.92; p = 0.01) [8]. It should be noted that for cases with a large lesion, it is possible that incomplete curettage can cause persistent inflammation due to the presence of residual tissues [42, 43]; hence, larger lesions usually heal more slowly [31]. This pattern was also noted in Lui et al.’s long-term study. They reported that the healing outcome of EMS was 71% at 1- to 2-years after apical surgery. Most healed cases maintained their healed status while those with uncertain healing gradually transitioned to complete healing over long-term follow-up, hence, their overall healing outcome improved to 78.3% at 5- to 9-years after apical surgery. Similarly, a long-term retrospective study [44] reported that EMS had a healing rate of 91.6% after one year and 91.4% after five years. However, these healing rates declined by 10% at 10 years to 81.5%. Therefore, long-term monitoring is needed to assess the outcomes of EMS.

The outcome of endodontic surgery can be influenced by the presence of a periodontal defect. [28, 45, 46]. Clinically, it is possible for a tooth to have endodontic and periodontal lesions that are independent of or communicate with each other. An isolated endodontic lesion typically has a closed wound with sinus tracts and a normal probing depth. A combined lesion may initially appear as an isolated endodontic or periodontal lesion with subsequent involvement of other lesions [47]. Therefore, a complete clinical history and accurate diagnosis are necessary for a successful outcome. Prior studies have shown that lesions that are solely of endodontic origin have a better chance of healing than those with an endodontic-periodontal origin [28–30]. Although statistically insignificant, our study confirmed this by demonstrating that the healing and outcome of EMS is better when lesions are solely of endodontic origin.

When considering periapical lesions and resorption of marginal bone, it is recommended that the buccal bone plate height be at least 3 mm [48]. In our study, although teeth with a longer height of buccal bone (> 3 mm) had better healing outcomes in EMS than those with a shorter height of buccal bone (≤ 3 mm), this difference did not significantly affect the outcome. This is most likely due to the wide variation in the sample sizes for the included studies, which leads to heterogeneous results [31, 39]. Alternatively, it could also be because the measurements were taken at a different time, specifically before and after the surgery. According to Kim et al. [39] the height of the buccal bone was measured preoperatively, but Song et al.[31] measured the height of the buccal bone postoperatively, which decreases after the ostectomy, leading to a difference in measurements.

Moreover, through and through lesions (TATL) present a unique clinical challenge, as the healing often results in periapical scarring, due to the growth of connective tissue into the defect [49]. Our study demonstrated that through and through lesions may have a slightly better outcome when a graft is placed. However, this result was statistically insignificant and there may be very little difference in healing of TATL, regardless of graft status at surgery.

There are some limitations to our systematic review. In terms of the risk of bias, the Cochrane Risk of Bias tool (Table 3) raised some concerns about performance and detection bias. As our included studies are a mix of prospective cohorts, retrospective cohorts, and randomized controlled trials, the differences in interventions and baseline characteristics may cause biases and discrepancies in the overall measurement. Our pooled analyses were based on a relatively wide range of subjects. The included studies primarily compared different hard tissue defects instead of evaluating the outcome of an individual defect, which negatively affected the domain of “overall certainty of evidence” of the quality assessment. Very low to low certainty indicates a potential discrepancy between the estimated and true outcomes. It is important to note that not all of the included studies reported long-term outcomes. Studies with short-term follow-up fail to account for the possibility of outcome relapse, and those that incorrectly classify incomplete healing outcomes as 'success' may inflate the actual success rate. Despite these limitations, our study has several strengths. We used a comprehensive search methodology with a strict inclusion criterion to maximize the amount of evidence. In addition, we analyzed the hard tissue defects and outcomes of EMS and used subgroup and sensitivity analyses to estimate the treatment effects.

Although endodontic research has increased, most studies report inconsistent measurements and outcomes. It is important to document relevant data consistently so that knowledge synthesis can occur over time, allowing healthcare providers to integrate their clinical expertise with the latest clinical research findings. To improve the reliability of endodontic outcome studies, the use of a reporting checklist [e.g. a 19-domain reporting checklist by Azarpazhooh et al. [50]] can address biases, unexplained heterogeneity, inconsistencies, indirectness, and imprecisions in overall quality assessment. It is important for future research studies to use this checklist as a guideline to ensure accuracy of the success rate in terms of EMS outcome. Larger sample sizes with minimal pre-operative confounders may also aid in obtaining a reliable outcome. Studies should document relevant data consistently so that knowledge synthesis can occur over time, allowing healthcare providers to integrate their clinical expertise with the latest clinical research findings. This will aid clinicians in the decision making process when considering EMS as a treatment option, specifically when there may be multiple hard tissue defects present pre-operatively.

## Conclusion

While many hard tissue characteristics were analyzed in this review, the results indicate that with a low to high quality of evidence, the size of the lesion appears to significantly influence the healing outcomes of EMS, with lesions ≤ 5 mm having a significantly better outcome than larger lesions. Additional hard tissue characteristics, such as the height of buccal bone < 3 mm, presence of a combined endodontic-periodontal lesion, and presence of through and through lesions, may also influence the outcome of EMS, but these results were statistically insignificant.

## Data Availability

The data that support the findings of this study are available from the corresponding author upon reasonable request.
